# Investigating singing imagery as an additional or alternative control task for EEG-based Brain-Computer Interfaces

**DOI:** 10.3389/fnhum.2025.1736711

**Published:** 2026-01-14

**Authors:** Hadi Mohammadpour, Sarah D. Power

**Affiliations:** 1 Department of Electrical and Computer Engineering, Faculty of Engineering and Applied Science, Memorial University of Newfoundland, St. John's, NL, Canada; 2 Division of Population Health and Applied Health Sciences, Faculty of Medicine, Memorial University of Newfoundland, St. John's, NL, Canada

**Keywords:** active brain-computer interface, electroencephalography, motor imagery, multi-class brain-computer interface, singing imagery

## Abstract

**Introduction:**

Brain-computer interfaces (BCIs) provide a movement-free means of communication and control, typically based on motor imagery (MI) tasks of hand, foot, or tongue movements. Most BCI studies focus on classifying up to four such tasks, which limits the number of available commands and restricts overall system functionality. Expanding the range of reliable mental tasks would directly increase the number of possible commands and thereby enhance the practical utility of BCIs. Singing imagery (SI) may offer an intuitive alternative or additional task to complement conventional MI paradigms.

**Methods:**

EEG data were recorded from 14 participants performing right-hand, left-hand, foot, and tongue MI, SI, and rest. Features were extracted using filter bank common spatial patterns (FBCSP), and tasks were classified with a random forest algorithm across 2-, 4-, 5-, and 6-class scenarios. Subjective data regarding participants' perceived task difficulty and general task preferences was also collected.

**Results:**

Classification accuracies with SI included were comparable to subsets of conventional MI tasks in 2-, 4-, and 5-class scenarios. In the 6-class scenario, average accuracy was approximately 60%, with six participants exceeding 70%, the level often cited as being necessary for effective BCI control. It is reasonable to expect performance to improve further with more advanced analysis methods and participant training.

**Conclusion:**

These promising results suggest that singing imagery can serve as both an additional and an alternative task in MI-BCIs. In lower-class systems, SI may provide a valuable option for generating commands, particularly for users who may find some conventional MI tasks less intuitive. When combined with the established MI tasks, SI could increase the number of possible commands, thereby extending the functional capacity of BCI systems. Overall, this work demonstrates the potential of SI to broaden the repertoire of mental tasks available for BCI control and to advance the development of more flexible, powerful, and user-centered BCI applications.

## 1 Introduction

Some individuals with severe physical disabilities may be unable to communicate and interact with their surroundings through the typical means of physical movements, speech, and gestures. Active brain-computer interface (BCI) technologies aim to provide an alternative pathway for communication and environmental control that does not rely on physical movement (Wolpaw and Wolpaw, 2012; Zander and Kothe, 2011). Active BCIs rely on intentionally generated brain patterns, whereas passive BCIs monitor cognitive or affective states without user intent, and reactive BCIs depend on stimulus-driven responses (e.g., SSVEP, P300). In an active BCI, users consciously produce distinct patterns of brain activity, which are then measured (using techniques like electroencephalography, or EEG), interpreted, and translated into commands for external devices. With an active BCI, a user could control a robotic arm (Looned et al., 2014), a wheelchair (Del R Millan et al., 2009; Yu et al., 2018), various computer applications (McFarland et al., 2008), or any number of other external devices.

For an accurate and efficient BCI design, it is crucial to elicit clear, distinct, and reproducible patterns of brain activity. These patterns are typically generated through the performance of specific mental tasks. Motor imagery (MI) is among the most widely used approaches: it can be performed kinesthetically, by imagining the sensations associated with movement, or visually, by imagining observing the movement; these two forms differ in both subjective experience and neural activation patterns (Kilintari et al., 2016). In active BCIs, kinesthetic MI of body parts is the most commonly used mental task, as it more reliably engages motor-related cortical areas. Indeed, the motor cortex, premotor cortex, supplementary motor area, and prefrontal regions are recruited during kinesthetic MI in ways that closely resemble their involvement in actual physical movement (Xu et al., 2019). In EEG-based MI-BCI systems, imagery of larger body parts - such as the hands, feet, and tongue - is frequently employed because the corresponding representations in the motor cortex cover relatively large cortical areas (Catani, 2017).

The number of differentiable commands is a crucial feature of an active BCI and plays a major role in determining the functionality and practicality of the system. Typically, BCI studies focus on investigating up to four specific tasks or commands including MI of the left hand (L), right hand (R), both feet (F), and tongue (T). It is desirable to expand the list of commands that a BCI system supports as this would allow users to have faster and more efficient communication and control, ultimately enhancing their overall experience. Indeed, increasing the number of supported commands has been identified as a significant remaining challenge in the field of MI-based BCIs (Yu et al., 2018). This challenge is compounded by obstacles such as low signal-to-noise ratio, limited spatial resolution of EEG signals, and user variability in MI performance, which have necessitated extensive user training and increased computational demands (Saha et al., 2021).

Classification of more than four tasks has been explored in a limited number of MI-BCI studies. Christensen et al. (2019) added a rest state to the conventional MI tasks (R, L, F, T) to create a 5-class online drone-control system, achieving an average accuracy of 41.8% across 10 participants. Other 5-class paradigms have been proposed by replacing tongue MI with simultaneous bilateral hand MI (Reshmi and Amal, 2013) or by combining single-limb MI with bilateral or limb-pair imagery (Kim et al., 2013), though these studies did not report clear performance metrics to assess functional usability. Faiz and Al-Hamadani (2019) demonstrated very high accuracy (97.5%) for imagined single-hand movements across five classes, but this result was obtained from only one participant, limiting its generalizability.

Beyond five classes, a few studies have used sequential MI paradigms in which multiple commands are inferred from combinations of simpler tasks (Yu et al., 2018; Jiang et al., 2015; Wang et al., 2011). For example, Jiang et al. (2015) generated six commands from sequential pairings of left-hand MI, right-hand MI, and rest, achieving high accuracies in a small sample. However, such multi-step paradigms inherently increase cognitive load, reduce information transfer rate, and may lead to user frustration. More recently, Huang et al. (2025) used MI of realistic 3D-object movements to build a six-class system, but achieved only 26% mean accuracy across seven participants.

Together, these studies illustrate that while multi-class MI-BCIs are technically feasible, many proposed approaches rely on tasks that are not intuitive, require uncommon or overly specific motor imagery, or involve multi-step sequences that are cognitively taxing. These limitations are particularly important when considering the target users of MI-BCIs since individuals with severe motor disabilities may find such tasks difficult to learn, sustain, or mentally differentiate. Thus, although progress has been made, there remains a need for multi-class paradigms that preserve classification performance while still being intuitive, user-friendly, and practical for long-term use.

While using MI tasks is most common, several EEG-based BCI studies have explored alternative non-motor mental tasks. Some common examples are mental calculation/counting (Myrden and Chau, 2015; Banville et al., 2017; Dutta et al., 2018; Sepulveda et al., 2007; Faradji et al., 2009; Friedrich et al., 2012; Scherer et al., 2013; Dobrea and Dobrea, 2009; Friedrich et al., 2011, 2013b,a; Obermaier et al., 2001; Friedrich et al., 2013c), word generation/association (Myrden and Chau, 2015; Banville et al., 2017; Dutta et al., 2018; Faradji et al., 2009; Friedrich et al., 2012; Scherer et al., 2013; Dobrea and Dobrea, 2009; Friedrich et al., 2011, 2013b,c), spatial navigation (Banville et al., 2017; Friedrich et al., 2012; Scherer et al., 2013; Friedrich et al., 2013b,a; Curran et al., 2004; Friedrich et al., 2013c), mental object rotation (Banville et al., 2017; Dutta et al., 2018; Faradji et al., 2009; Friedrich et al., 2012; Scherer et al., 2013; Dobrea and Dobrea, 2009), and facial imagery (Friedrich et al., 2012; Scherer et al., 2013). A majority of these studies focused on binary classification between pairs of tasks, however some of them explored 4-class classification (Dobrea and Dobrea, 2009; Friedrich et al., 2011, 2013a; Obermaier et al., 2001) and one examined the feasibility of a 5-class BCI (Obermaier et al., 2001). In this 5-class study, the possibility of incorporating a mental calculation task alongside the four common MI tasks (L, R, F, T) was investigated, and an average accuracy of 53% across three participants was achieved (Obermaier et al., 2001). Unfortunately, many of these non-motor tasks are unnatural, unintuitive, or difficult to perform, and even when they yield high accuracies in healthy participants, they may not translate into user-friendly, long-term solutions for the target population.

Singing imagery (SI) is an intuitive task that, despite its potential advantages, has not yet been thoroughly explored for developing active BCIs. SI is the simple task of singing a song in one's head and is a common experience for most people. Because SI draws on articulatory-motor imagery rather than imagery of gross limb movements, it may be more accessible for individuals with severe motor impairments. SI may also hold particular promise for individuals who retain musical or melodic processing despite diminished linguistic communication, such as some patients with dementia (Baird and Samson, 2015). In addition, SI may be well suited not only for discrete commands but also for continuous, sustained actions (e.g., scrolling through content or moving a video game avatar forward). Most other mental tasks - both motor and non-motor - are difficult to maintain for more than a few seconds and would require repeated start/stop commands for extended actions. In contrast, simply initiating and continuing SI to sustain an action until reaching the desired endpoint may be relatively easy for many users.

Some EEG-based BCI studies have investigated SI, but only in binary classification scenarios as part of a larger set of mental tasks. In these studies, SI generally produced lower accuracies than other tasks (Myrden and Chau, 2015; Banville et al., 2017). However, this may be attributable to how SI was defined. In one case, participants were simply instructed to “sing a song of their choice in their heads” (Myrden and Chau, 2015), and in another they were asked to “imagine singing a song they chose beforehand, preferably with lyrics, while focusing on the emotional response it elicits” (Banville et al., 2017). SI may elicit clearer, more discriminable neural responses when it is framed as 1) a form of speech imagery involving articulatory preparation, motor planning, and auditory activation (Panachakel and Ramakrishnan, 2021), and/or 2) a motor imagery task involving imagined movements of the jaw, lips, and tongue. Prior work on covert or imagined speech typically shows involvement of classical language regions together with orofacial motor and somatosensory cortices, suggesting that explicitly imagining the lyrics rather than only the tune may be key to producing strong, class-separable activation patterns. Notably, earlier SI studies did not instruct participants to focus on articulatory or lyric-based imagery, which may partly explain the lower classification accuracies previously reported. Of course, SI likely engages a broader set of cognitive and neural processes beyond articulatory-motor and auditory mechanisms, possibly including memory retrieval, rhythmic timing, musical imagery, and affective or reward-related responses associated with familiar songs.

Although SI showed relatively lower classification performance in Banville et al. (2017), it was rated as having the lowest mental demand, effort, and frustration among all tasks. Given these potential advantages, a more comprehensive investigation of SI is warranted. In particular, examining SI with explicit attention to its speech- and motor-imagery components, and evaluating it in both binary and multi-class settings, may provide a clearer assessment of its effectiveness as a reliable and user-friendly BCI control task.

It should be noted that our framing of SI in this study is fundamentally different from typical “speech imagery” BCI paradigms. In speech imagery BCIs, the objective is to decode linguistic content (e.g., words, phonemes), whereas here SI is used simply as an intuitive mental task that reliably produces a distinct neural pattern suitable for use as an additional BCI command. While covert speech could likely serve a similar purpose, SI was chosen because it provides a structured, rhythmic articulatory sequence that many users find easier to sustain than covert speech, while also allowing control over important task parameters such as familiarity and tempo. SI is therefore not presented as an alternative to speech decoding, but as a practical, intuitive articulatory-motor imagery task for multi-class BCI control.

In this study, taking into consideration the commonly used MI tasks in EEG-based BCI research (i.e., R, L, F, T, REST), we explored the potential of SI in binary and multi-class scenarios. Our objectives were: (1) to assess the potential of SI as an alternative to conventional MI tasks in 2-, 4-, and 5-class paradigms (i.e., evaluating whether SI can provide similar levels of performance), and (2) to assess the potential of using SI along with the conventional MI tasks to increase the number of available commands from five (i.e., L, R, F, T, REST) to six (i.e., L, R, F, T, REST, SI).

This work is described in detail in the following sections. Section II describes the experimental procedures and analytical methods used to collect and process the EEG and subjective data provided by the participants. This includes the EEG preprocessing steps, feature extraction and selection, and classification approaches, as well as the analysis of the subjective responses. Section III presents the main results, including classification performance across task types and insights from the subjective evaluations. Section IV discusses the implications of these findings, limitations of the study, and directions for future research. Section V concludes the paper by summarizing the key contributions and highlighting the potential of SI as a BCI control strategy.

The work described in this article was reported in the Master's thesis of one of the authors (H.M.) (Mohammadpour, 2023).

## 2 Methodology

### 2.1 Participants

Fifteen healthy participants (mean age: 25±5.8 years; 12 right-handed; 10 female) took part in this study. Participant inclusion criteria required having normal or corrected-to-normal vision and hearing, and not having any cognitive impairment and/or history of neurological diseases, disorders, or injuries. For at least four hours prior to the experiment, participants were instructed to refrain from exercising and consuming alcohol or caffeine. Written informed consent was obtained from all participants prior to their session, and the experimental protocol was approved by the Interdisciplinary Committee for Ethics in Human Research (ICEHR) at Memorial University of Newfoundland. Data collected from one participant who displayed a lack of engagement and significant drowsiness during the experiment were excluded from the analysis.

### 2.2 Data acquisition

A 64-channel actiCHamp system equipped with active electrodes (Brain Products, GmbH) was used for data collection. The sampling rate was 500 Hz. The impedance for all electrodes, which were placed according to the international 10-10 system, was maintained below 10 KOhm throughout the experimental session. Markers indicating the timing of different mental tasks were transmitted via the TriggerBox (Brain Products, GmbH) to the recording software (Brain Vision Recorder).

### 2.3 Experimental protocol

**Figure 1:**
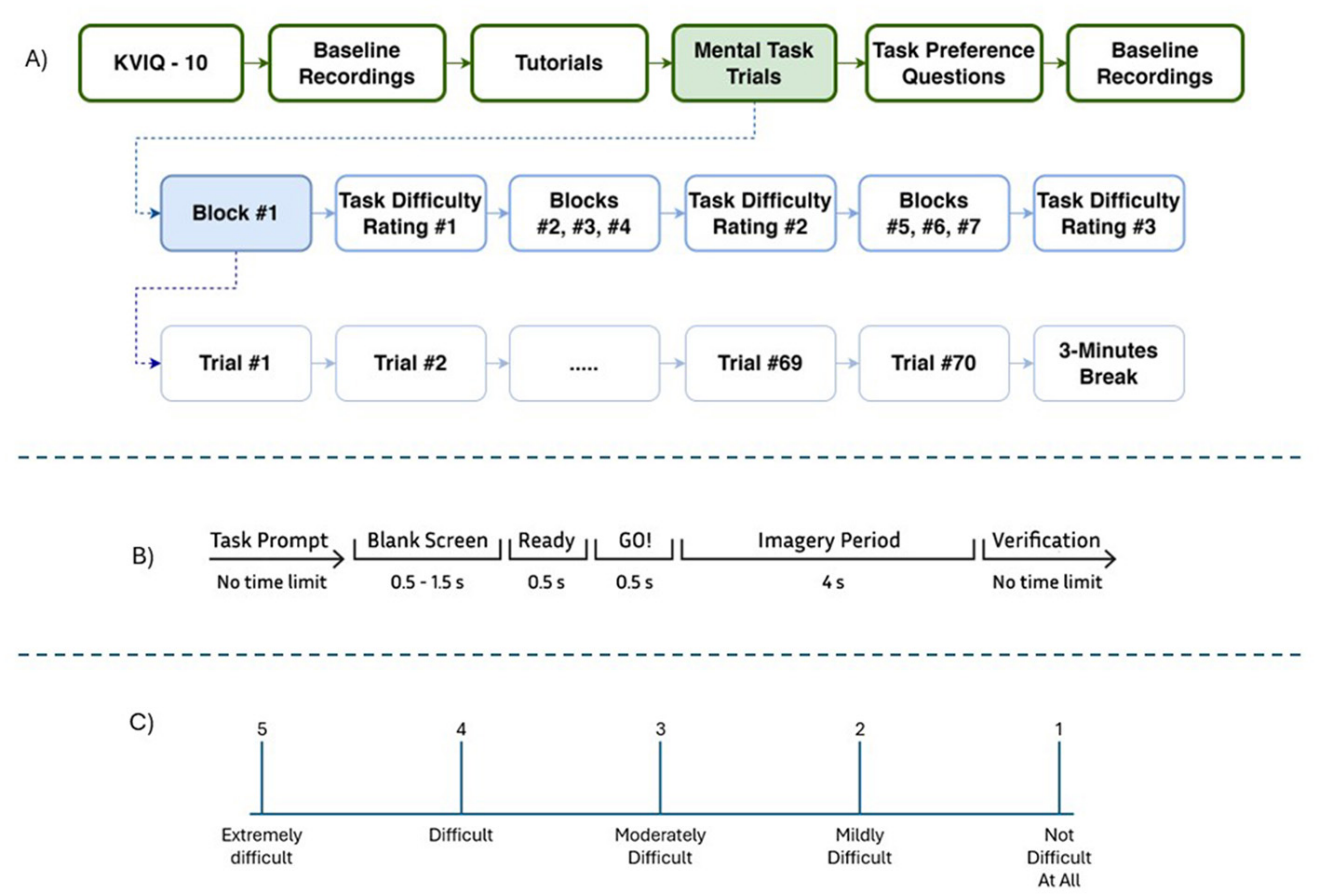
**(A)** Experimental protocol illustrating the different steps of the single recording session. All of the blocks had a similar structure to Block #1. **(B)** Timing of the individual trial. **(C)** Task difficulty rating scale.

Each participant completed a single experimental session, which is outlined in Figure 1A. The details of the protocol are described below. Psychtoolbox in MATLAB was used to develop a graphical user interface that guided users from one step to the other. The tutorial, mental task cues, task difficulty ratings and task preference questions were all presented via this simple interface. This interface also sent event markers to the recording software via the TriggerBox (Brain Products, GmbH) to synchronize the data with the timing of the different mental tasks.

Before starting the main experiment, participants were asked to complete the short version of the Kinesthetic and Visual Imagery Questionnaire (KVIQ-10) (Malouin et al., 2007), which was also presented through the experimental interface. KVIQ-10 is a standardized questionnaire that is commonly used in MI-BCI studies to assess participants' ability to visualize and sense imagined movements. Collecting KVIQ-10 ratings could potentially help in the interpretation of individual participants' classification results.

Next, participants were seated in a comfortable chair in front of a standard computer monitor and were fitted with the EEG electrodes. Then, two one-minute baseline trials were recorded, one with the participant's eyes open and the other with their eyes closed. Two more such baseline trials were collected at the end of the experiment as well. Before beginning the imagery trials, a detailed description of the experimental protocol and the specific imagery tasks was provided to the participants to ensure they had a clear understanding of the tasks they were expected to perform.

A total of seven different mental tasks, including a rest state, were performed by the participants. The specific details and descriptions of these tasks can be found in Section 2.3.1. 70 trials were completed for each task, resulting in a total of 490 trials. These trials were divided into seven blocks, with each block consisting of 70 trials (10 randomly dispersed trials per task per block, with a different order for each block). Participants were required to have a minimum of 3 minutes of rest between blocks to minimize fatigue and to help maintain their focus throughout the experiment.

Figure 1B illustrates the timing sequence for each trial. Each trial had a duration of 5.5–6.5 s. Before each trial began, the task the participant was to perform was displayed on the screen. When the participant felt ready to begin, they pressed any key on the keyboard to initiate the timed portion of the trial. Once a key was pressed, a blank screen was presented for a random period between 0.5 and 1.5 s, drawn from a uniform random distribution over the specified interval. This was followed by a “Ready!” and then a “GO!” screen, each of which appeared for 0.5 s. Following that, a dark screen with only a “+” sign was displayed for a duration of 4 seconds, indicating the period during which the participant was required to perform the specified mental task. The final step of each trial was the verification step, which asked participants to provide feedback on their own performance during each trial. Participants selected one of the following three options to indicate their perception of the quality of their task performance in the previous trial: (1) I performed it correctly, (2) I did not perform it correctly, and (3) I performed it correctly but not very well. Participants selected the first option if they performed the indicated task correctly and felt their performance was strong. On the other hand, they chose the second option in case they did not perform the task at all, performed the wrong task, or made unintentional movements during the task period. Lastly, the third option was for cases where participants did the task correctly but felt their performance was weak due to lack of focus or engagement. The purpose of this step was to gather subjective feedback from participants about their own performance during the task trials, allowing for a more comprehensive interpretation of the results.

#### 2.3.1 Mental tasks

In addition to the four conventional MI tasks (right hand, left hand, both feet, and tongue), we incorporated a resting state (REST) and two distinct SI tasks (SI_Kin_ and SI_noKin_). The tasks were defined as follows:

- **Right hand MI (R):** Participants imagined tapping their right hand, bending at the wrist, at a steady pace of approximately one tap per second.
- **Left hand MI (L):** Participants imagined tapping their left hand, bending at the wrist, at a steady pace of approximately one tap per second.
- **Feet MI (F):** Participants imagined tapping both feet together, bending at the ankle, at a steady pace of approximately one tap per second.
- **Tongue MI (T):** Participants imagined protruding their tongue out of their mouth and then retracting it at a steady pace of approximately once per second.
- **Kinesthetic SI (SI**_Kin_**):** Participants imagined singing a song (with lyrics) in their heads, without vocalization or movement. In doing so, participants were instructed to focus on the kinesthetic sensation of moving their jaw, tongue, and lips as they imagined articulating the lyrics.
- **Non-Kinesthetic SI (SI**_noKin_**):** Participants imagined singing a song (with lyrics) in their heads, without vocalization or movement, and without focusing on the kinesthetic sensations in the jaw, tongue and lips.
- **Rest (REST):** Participants were instructed that they do not need to do anything specific for this task. They were asked to keep their eyes open and focused on the screen (they could blink normally) and not perform any of the other six tasks in this experiment.

As mentioned earlier, we consider SI to be a unique form of speech imagery and/or a potentially more intuitive and natural MI task involving the movements of the jaw, tongue, and lips. Aiming to highlight these aspects of SI and explore whether one version shows more potential as a control task for BCIs, we included the two different SI tasks described above. For the SI tasks, participants were asked at the start of the experiment to select seven songs that were very familiar to them from a provided list of well-known English-language songs. All songs on the list featured simple lyrics and a similar upbeat tempo (e.g., “Jingle Bells,” “Happy Birthday,” “The Alphabet Song”). At the beginning of each block, participants were told which of their selected songs to use for that block, resulting in one song per block across the seven blocks of the experiment. The same song was used for both SI tasks (SI_Kin_ and SI_noKin_) within a given block to avoid confounding song with task type. Participants were free to imagine any lyric-containing portion of the chosen song during the imagery periods. These constraints were intended to minimize variability in SI performance due to differences in song familiarity or tempo. Using a different song in each block ensured that the classifier learned neural patterns associated with the general task of SI rather than with imagining a specific song.

#### 2.3.2 Subjective evaluation of tasks

To gain deeper insights into participants' subjective experience with the imagery tasks, we collected both difficulty ratings and post-experiment task preferences. After blocks 1, 4, and 7, participants rated the difficulty of each task on a scale from 1 (“not difficult at all”) to 5 (“extremely difficult”), as shown in Figure 1C.

After completing all seven blocks, participants answered three multiple-choice questions designed to probe different aspects of task preference and usability:

**1) Which four tasks would you choose to use on a daily basis to work with a computer?** This question assessed overall preference across all six tasks (REST was not included), providing a general indication of which tasks participants found most practical or appealing for daily BCI use.
**2) Which task would you prefer to use in a BCI on a daily basis? (T or either SI)** This question was limited to tongue MI and SI because both involve imagery of the mouth and jaw. For smaller BCI command sets, we envisioned these two orofacial tasks as potential alternatives to one another, making a direct preference comparison useful. This does not preclude using both in larger multi-class settings, as reflected in our 6-class analysis.
**3) Which singing task did you find more intuitive and easier to perform? (SI**_noKin_ **or SI**_Kin_**)** This question compared SI_noKin_ and SI_Kin_ to determine which variant participants felt was more intuitive, providing insight into which version may be more suitable for practical BCI implementation.

### 2.4 EEG preprocessing

Using the EEGLAB toolbox in MATLAB, data was first down-sampled to a frequency of 250 Hz, then re-referenced to the average of all channels. Epochs were then extracted and baseline correction was performed (i.e., the mean value of each epoch was subtracted). The epochs were extracted for the period of −500 ms to 4,000 ms from the onset of the imagery portion of each trial.

We note that more advanced preprocessing pipelines, including online artifact removal methods such as artifact subspace reconstruction (ASR) (Mullen et al., 2015), are increasingly feasible in real-time BCIs. Our decision to use the lightweight preprocessing pipeline described above reflects our aim of evaluating task separability under conservative, minimally processed conditions. Because all features were restricted to the 4–40 Hz range (see Section 2.5), line noise (60 Hz) and high-frequency EMG components fall outside the analyzed bands. Although ocular artifacts have most of their power below ~4 Hz, they can extend slightly into the lower end of the analyzed range; however, any such residual activity would be expected to manifest as nonspecific noise rather than structured, task-related activity.

### 2.5 Feature extraction and selection

The Filter Bank Common Spatial Patterns (FBCSP) algorithm (Ang et al., 2012) was used for feature extraction. FBCSP is widely recognized as one of the most effective feature extraction methods for MI-BCI (Lotte et al., 2018). The details of the features used in our analysis, based on the FBCSP algorithm, are provided below.

In this study, nine frequency bands were extracted from each EEG epoch using symmetric linear-phase FIR bandpass filters. The filters covered the range from 4 Hz to 40 Hz, with non-overlapping passbands of 4 Hz bandwidth (i.e., 4–8 Hz, 8–12 Hz, 12–16 Hz, 16–20 Hz, 20–24 Hz, 24–28 Hz, 28–32 Hz, 32–36 Hz, 36–40 Hz). The CSP algorithm was then applied separately to each of the nine frequency bands, yielding 10 optimized CSP filters (5 pairs) per band. The 64-channel EEG epochs were projected onto these filters, and the log-variances of the resulting components from each frequency band were extracted as input features. A total of 90 features were extracted (10 spatial filters × 9 frequency bands). Spectral features in the 4–40 Hz range were selected because μ-β rhythms reliably reflect motor-related and articulatory-motor processes, which are engaged not only during limb motor imagery but also during imagined speech and singing. Using the same FBCSP-based spectral features for all tasks ensured that MI and SI were evaluated within a consistent and physiologically grounded feature space. As both binary and multiclass classification problems were explored, the multiclass CSP implementation from the MNE-Python library was used, which extends CSP to problems involving more than two classes following the approach proposed in Grosse-Wentrup and Buss (2008).

**Figure 2:**
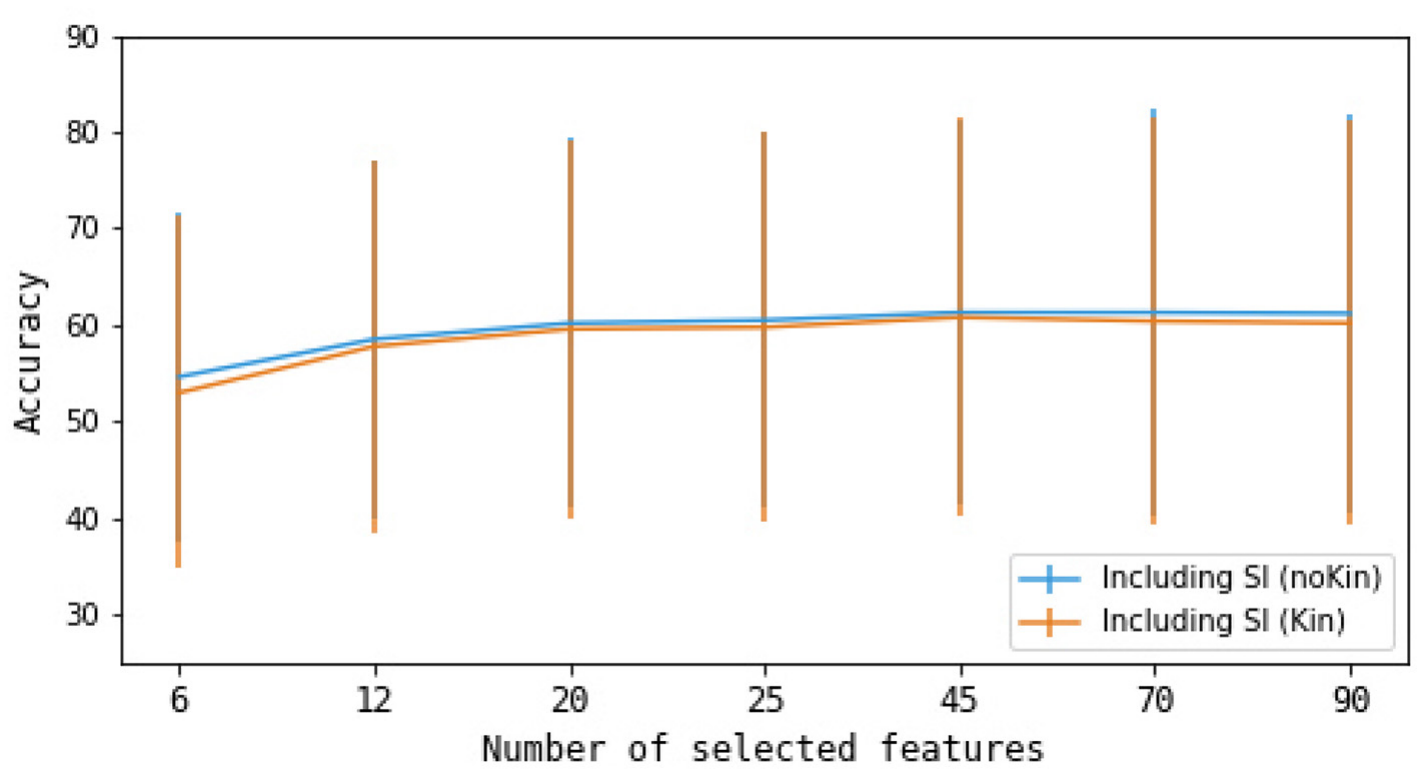
Mean classification accuracies for the 6-class scenarios for different numbers of selected features. Error bars represent the standard deviation across participants.

Next, we applied the Maximum Relevance Minimum Redundancy (mRMR) feature selection algorithm (Peng et al., 2005) to reduce the dimensionality of the feature set for all classification problems considered (described in the next section). This algorithm selects features that have high relevance to the target variable (in this case, the task labels) and low redundancy with respect to each other. Mutual information is used to assess relevance (between each feature and the class) and redundancy (between candidate and already-selected features) separately. These are then combined into a single selection criterion; here, the Mutual Information Difference (MID) formulation was used. We implemented mRMR using the pymrmr Python library, selecting the top 25 features. This choice was based on preliminary analysis of the 6-class classification problems, where we examined feature sets of various sizes, including 6, 12, 20, 25, 45, 70, and 90. We observed that the accuracy gains reached a plateau after approximately 25 features (see Figure 2). Hence, 25 features were deemed sufficient for our purposes.

### 2.6 Task classification via EEG

The main objectives of this study were to explore the potential of SI as (1) an alternative to conventional MI tasks in 2-, 4- and 5-class paradigms, and (2) as a means for expanding the number of available commands from five (L, R, F, T, REST) to six (L, R, F, T, REST, SI). To achieve this, we first examined all possible combinations of the seven tasks (L, R, F, T, REST, SI_noKin_, SI_Kin_) in 2-, 4-, and 5-class scenarios to evaluate whether accuracies obtained for combinations with SI are comparable to those that do not incorporate SI. Moreover, two separate 6-class scenarios were examined where one of the two SI tasks was added to the combination of conventional MI tasks and REST. It should be noted that for scenarios beyond the 2-class analysis, combinations including both SI_noKin_ and SI_Kin_ were excluded.

A random forest classifier was implemented using the Python *Scikit-learn* library, with all hyperparameters set to their default values. Specifically, the model comprised 100 decision trees, with splits determined by the Gini impurity criterion (Breiman et al., 1984). To avoid restricting model complexity a priori, the maximum depth of each tree was left unconstrained, allowing growth until all leaves were pure or contained fewer than two samples for splitting. Bootstrap sampling was employed, and the number of features considered at each split was set to the square root of the total number of features, following standard practice. The random forest classifier was selected because it is robust to noise, can capture nonlinear decision boundaries, and performs well with the high-dimensional feature spaces like that generated by filter bank CSP.

For each classification problem, classifier performance was estimated as the average across five runs of 10-fold cross-validation. In each fold, only the training data were used to optimize the CSP filters and train the classifier, while the test data were reserved exclusively for performance evaluation.

**Figure 3:**
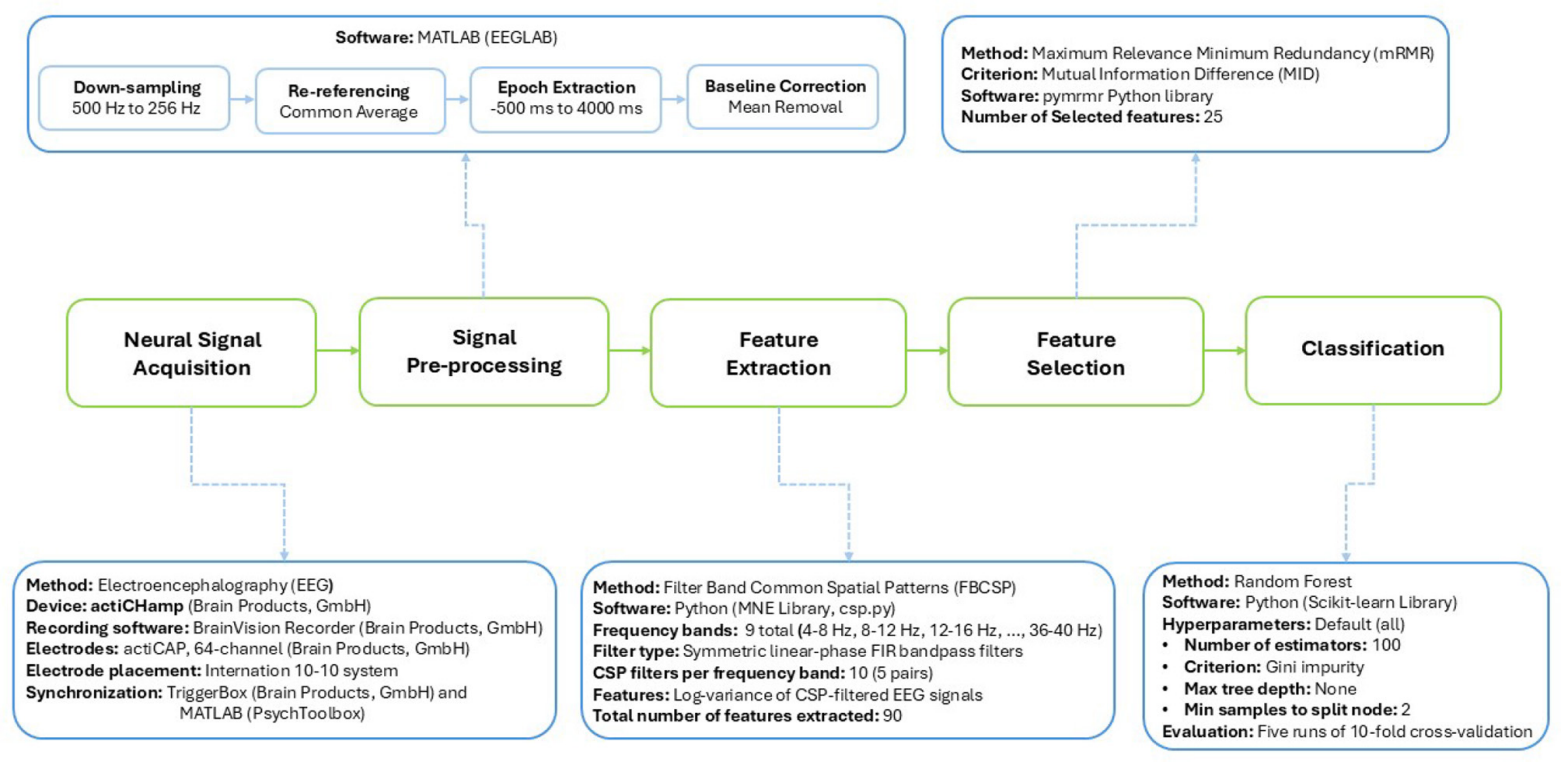
EEG data classification pipeline.

The full EEG data classification pipeline and relevant details of each step are summarized in Figure 3.

#### 2.6.1 Statistical analysis

To assess whether the observed classification accuracies exceeded chance, we computed analytic significance thresholds for each classification scenario using the binomial cumulative distribution method described in Combrisson and Jerbi (2015). Because empirical chance levels can exceed theoretical chance accuracy in finite-sample settings, this approach provides a more appropriate benchmark than relying on theoretical chance alone.

For the binary, 4-class, 5-class, and group-level 6-class analyses, we calculated the accuracy threshold corresponding to *p* = 0.05 for the number of classified trials in each scenario. The mean accuracies were then compared to these thresholds to determine whether, on average across participants, classification accuracies exceeded this benchmark.

For the per-participant results in the 6-class scenario, we additionally performed a permutation-based significance test. Unlike the other scenarios, which include many different task combinations, the 6-class configuration consists of only two fixed combinations and was therefore a tractable setting for a more computationally intensive resampling procedure. For each participant, the class labels were randomly shuffled and the full classification pipeline was re-run to generate a distribution of chance-level accuracies. Each participant's true-label accuracy was then compared to their corresponding shuffled-label distribution using a paired *t*-test.

### 2.7 Self-evaluation data

#### 2.7.1 Effect of discarding potentially “bad” data on classification accuracy

Secondary analysis involved investigating whether the participants' self-evaluation at the end of each trial (i.e., the responses to the verification step) could be an indicator of “bad” data that should be excluded. This analysis was applied to the 6-class scenario, which represents the most demanding classification setting and contains the largest number of self-reported bad trials, making it the condition in which any potential benefit of excluding low-quality trials would be most apparent. Based on the responses obtained during the verification step of each trial, we repeated only the 6-class classification problems under two independent scenarios, as follows: (1) After removing only trials where participants responded with “I did not perform it correctly,” and (2) After removing trials where participants responded with either “I did not perform it correctly” or “I performed it correctly but not very well.” For each of these scenarios, the results were compared to those obtained when an equivalent number of trials was randomly removed from each participants' dataset. This random removal ensured that the sizes of the training sets remained identical across the different conditions and did not affect the performances obtained in the different scenarios. Comparing the performance in these different scenarios could indicate whether participant self-evaluation of task performance is a reliable indicator of the “quality” of the underlying brain patterns.

#### 2.7.2 Correlation analyses

- Self-evaluation and classification accuracy

We examined the correlation between participants' self-evaluation ratings and the 6-class accuracies obtained when no trials were excluded. More specifically, we calculated the correlation between the 6-class classification accuracy and the percentage of trials in which participants responded with (1) “I performed it correctly,” and (2) either “I performed it correctly” or “I performed it correctly but not very well.”

- KVIQ-10 scores and classification accuracy

We also explored the correlation between participants' MI ability, assessed through their KVIQ-10 scores, and the 6-class accuracies obtained when no trials were excluded. More specifically, we calculated the correlation between the 6-class classification accuracy and the participants' (1) visual imagery, (2) kinesthetic imagery, and (3) overall KVIQ-10 scores.

- Self-evaluation and KVIQ-10 scores

The correlation between participants' KVIQ-10 scores and their self-evaluation of task performance was also examined. Specifically, the correlation was calculated between the KVIQ-10 scores and the percentage of trials in which participants indicated (1) “I performed it correctly,” and (2) either “I performed it correctly” or “I performed it correctly but not very well.”

## 3 Results

### 3.1 Participant task preferences and difficulty ratings

**Figure 4:**
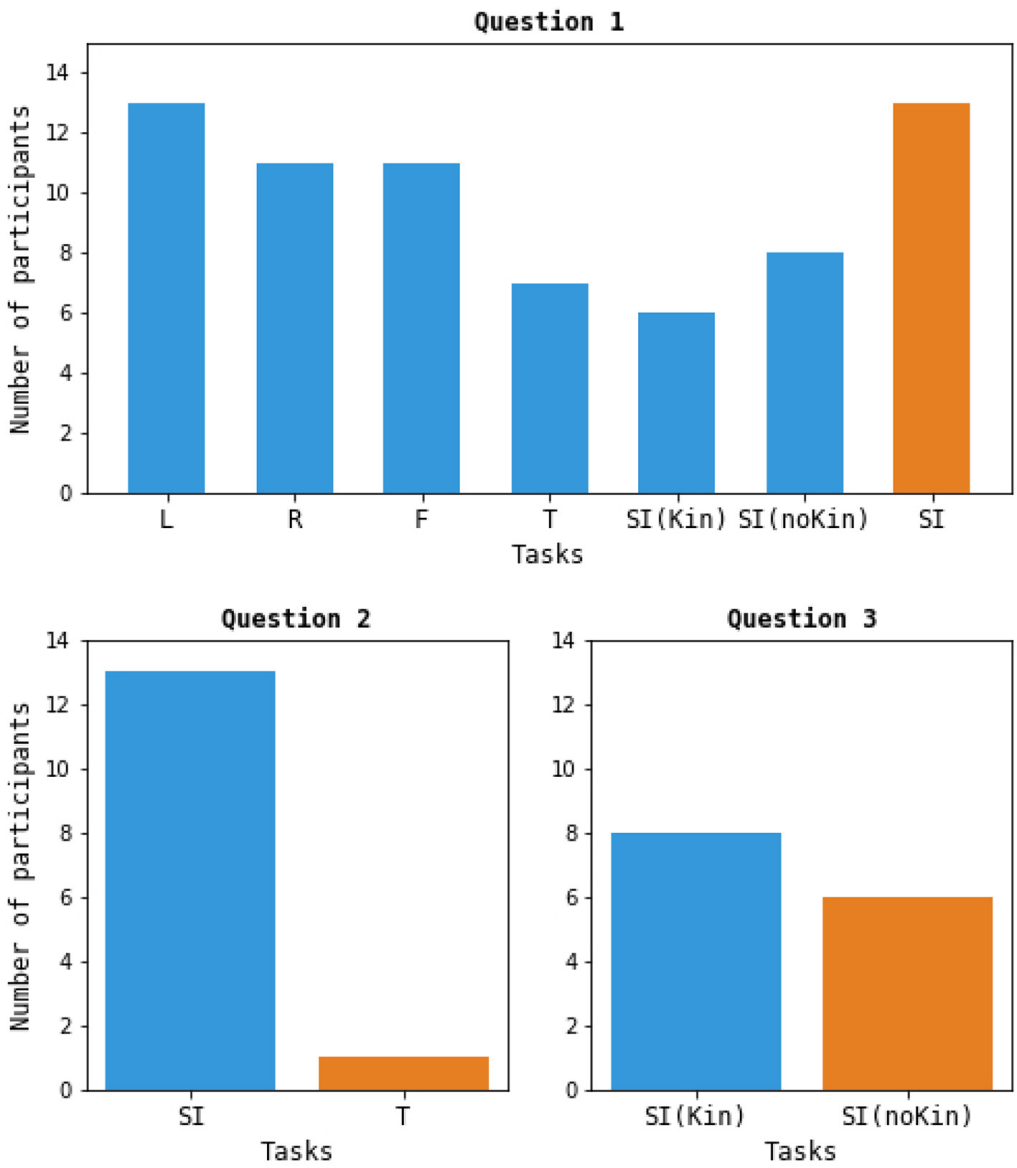
Participant responses to the task preference questions. In Question 1, the results shown for “SI” reflect all participants who included either one of the two SI tasks as one of their top four choices.

The participants' responses to the question “Which four tasks would you choose to use on a daily basis to work with a computer?” is summarized in Figure 4. The number of participants who included each task as one of their top four choices is shown. Figure 4 also shows the responses to the other two questions regarding task preferences: 13 out of 14 participants expressed a preference for the SI tasks over the tongue MI task, and 8 out of 14 participants indicated a preference for SI_Kin_ compared to SI_noKin_.

The participants rated their perceived difficulty of each task on a five-point scale at three points during the experiment: after finishing Blocks 1, 4, and 7. The difficulty ratings for each task, averaged across all participants, are presented in Table 1.

**Table 1:** Task difficulty ratings, averaged across participants, for each of the seven mental states.

	**Recording after**			
	**Block 1**	**Block 4**	**Block 7**	**Task average**
L	2.1 (1.0)	1.9 (0.7)	1.9 (0.9)	2.0 (0.8)
R	2.2 (0.8)	2.0 (0.8)	1.9 (0.9)	2.1 (0.7)
F	1.9 (0.8)	2.0 (1.2)	1.9 (1.1)	2.0 (1.0)
T	2.9 (1.4)	2.2 (1.1)	2.2 (1.2)	2.4 (1.1)
SI_Kin_	2.6 (1.5)	2.4 (1.2)	2.4 (1.4)	2.5 (1.3)
SI_noKin_	2.2 (1.3)	1.9 (0.8)	1.8 (0.8)	2.0 (1.0)
REST	1.6 (1.0)	1.4 (0.7)	1.2 (0.4)	1.4 (0.9)
Block average	2.2 (0.6)	2.0 (0.6)	1.9 (0.6)	

Standard deviations appear in parentheses.

### 3.2 EEG classification

The outcomes of the 2-class, 4-class, 5-class, and 6-class classification scenarios are presented in Tables 2–5. Each table reports the grand average accuracy across all participants (with standard deviations in parentheses) for every possible task combination, with the exception of combinations involving both SI task variants, which were examined only in the binary scenario. Combinations including only the four traditional MI tasks and REST are also provided to serve as benchmarks for evaluating combinations that include SI. In the 2-class analyses, the SI_Kin_ vs. SI_noKin_ accuracy was excluded from the average-accuracy calculation for each task.

**Table 2:** Classification accuracies for the 2-class scenarios (%).

**2-class scenarios**							
**Statistical significance threshold** = **57.1%**							
	**Conventional motor tasks**			**Singing imagery**			
	**R**	**F**	**T**	**SI**_Kin_	**SI**_noKin_	**REST**	**Average**
L	81.7 (15.0)	86.7 (13.7)	89.9 (9.5)	90.0 (9.0)	90.5 (8.3)	87.5 (10.4)	87.7 (3.3)
R	-	87.2 (13.3)	89.2 (9.5)	88.7 (10.2)	88.2 (9.3)	85.6 (10.9)	86.8 (2.8)
F	-	-	82.6 (14.2)	84.5 (12.7)	85.9 (11.3)	83.6 (12.1)	85.1 (1.9)
T	-	-	-	71.8 (12.8)	78.9 (13.5)	82.7 (11.2)	82.5 (6.7)
SI_Kin_	-	-	-	-	67.6 (14.2)	83.1 (12.9)	83.5 (7.2)
SI_noKin_	-	-	-	-	-	74.1 (12.1)	83.6 (6.8)
REST	-	-	-	-	-	-	82.8 (4.6)

**Table 3:** Classification accuracies for the 4-class scenarios (%).

**4-Class scenarios**			
**Statistical significance threshold** = **29.3%**			
L + R + F + T	72.8 (18.3)		
30.5-1,15.2	**Singing imagery**		
	**SI**_Kin_	**SI**_noKin_	**REST**
L + R + F +	73.7 (18.4)	73.7 (19.0)	72.6 (19.0)
R + F + T +	67.6 (17.1)	70.0 (18.0)	70.0 (17.7)
L + F + T +	66.6 (16.8)	70.2 (18.1)	69.5 (19.0)
R + L + T +	69.0 (16.5)	73.3 (17.0)	72.5 (17.8)
F + T + REST +	62.0 (17.3)	63.1 (15.8)	-
R + T + REST +	66.0 (15.4)	67.5 (14.1)	-
R + F + REST +	70.6 (18.0)	68.3 (15.5)	-
L + T + REST +	67.0 (15.5)	68.0 (14.6)	-
L + F + REST +	71.4 (17.9)	68.1 (15.4)	-
R + L + REST +	71.9 (18.0)	69.8 (15.6)	-

**Table 4:** Classification accuracies for the 5-class scenarios (%).

**5-class scenarios**		
**Statistical significance threshold** = **23.3%**		
L + R + F + T + REST	66.5 (20.1)	
30.5-1,15.2	**Singing imagery**	
	**SI**_Kin_	**SI**_noKin_
L + R + F + T +	63.8 (18.8)	67.1 (19.8)
L + F + T + REST +	62.1 (18.3)	62.1 (18.0)
R + F + T + REST +	62.9 (17.6)	62.1 (18.0)
L + R + T + REST +	63.6 (17.7)	64.6 (17.7)
L + R + F + REST +	66.9 (20.3)	65.5 (18.0)

**Table 5:** Classification accuracies for the 6-class scenarios (%).

**6-Class scenarios**		
**Statistical significance threshold** = **19.8%**		
	**Singing imagery**	
	**SI**_Kin_	**SI**_noKin_
L + R + F + T + REST +	59.7 (19.7)	60.7 (19.2)

Tables 2–5 also list the binomial chance thresholds for each scenario, derived using the binomial cumulative-distribution method described in the Statistical Analysis section.

**Figure 5:**
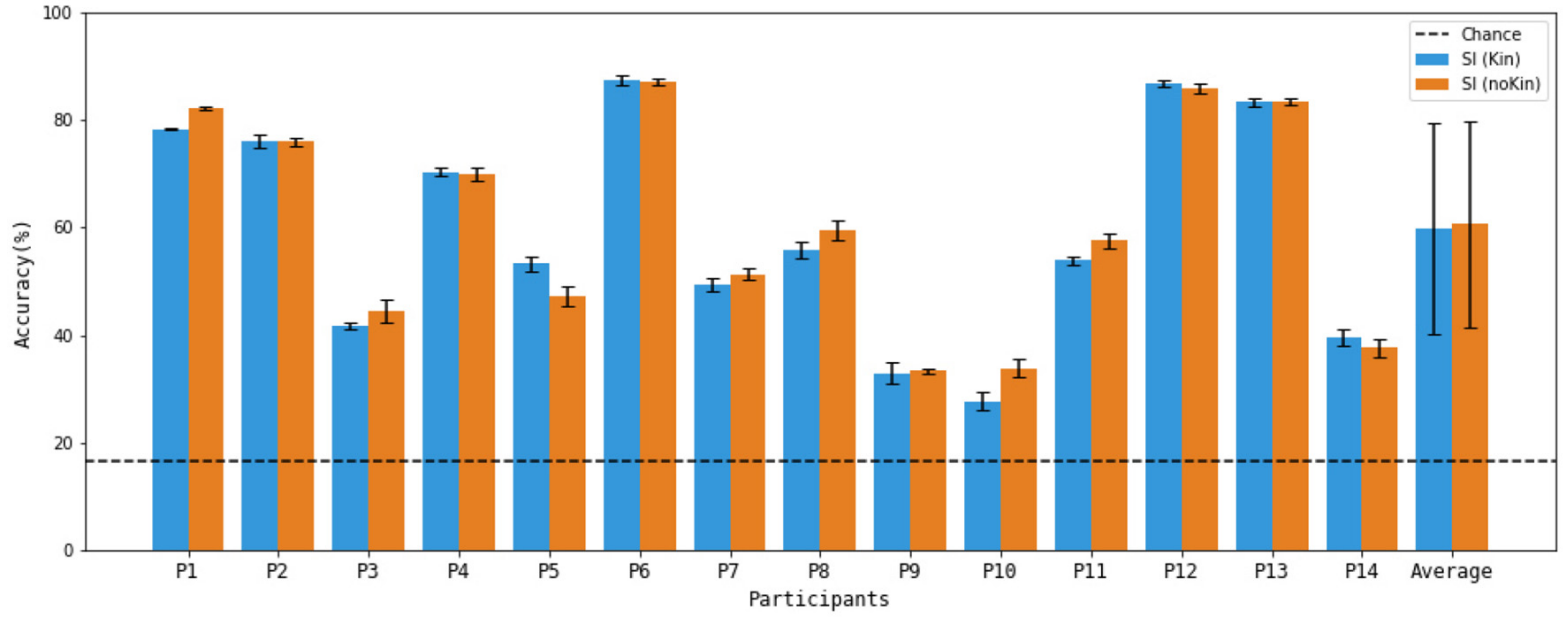
6-class classification accuracies achieved for all the participants in this study.

Figure 5 shows the per-participant accuracies for the two 6-class configurations. As detailed in the Statistical Analysis subsection, statistical significance for these participant-level results was assessed using permutation-based chance distributions. Paired *t*-tests indicated that all participants achieved significantly above-chance performance in both 6-class scenarios (*p* < 0.001). The average permutation-based chance accuracies were 16.7 ± 0.8 and 19.6 ± 0.9 for the SI_Kin_ and SI_noKin_ combinations, respectively.

**Figure 6:**
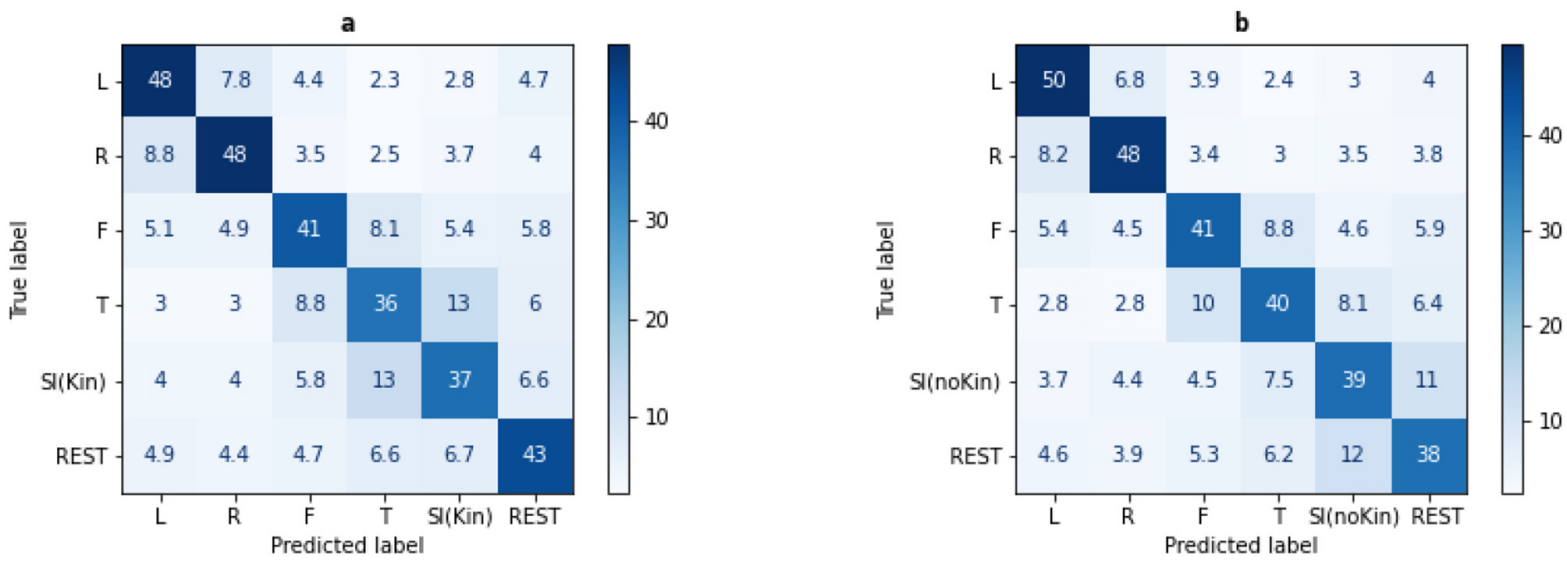
Confusion matrices for the two 6-class scenarios. **(a)** Combination with SI_Kin_ **(b)** Combination with SI_noKin_. Results of P8, which only had four blocks of data (and, as a result, a different total number of trials), were excluded in the average confusion matrices.

Figure 6 presents the average confusion matrices for the two 6-class configurations (conventional tasks + SI_Kin_, and conventional tasks + SI_noKin_), illustrating the distribution of class-specific prediction performance.

### 3.3 Self-evaluation data

**Figure 7:**
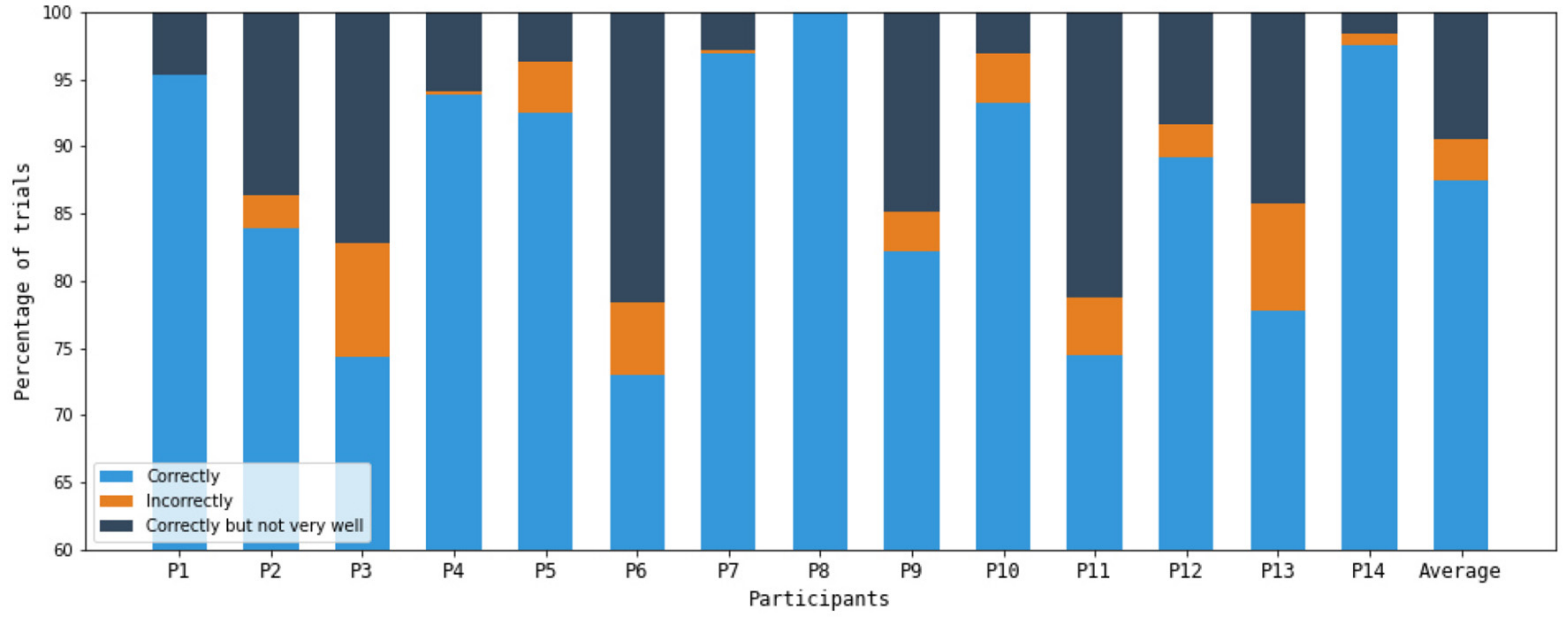
Results of self-evaluation for all of the participants.

Figure 7 presents the self-evaluation results for each participant. The figure shows the percentage of trials in which participants indicated they performed the task “correctly,” “not correctly,” or “correctly but not very well.”

#### 3.3.1 Effect of discarding potentially “bad” data on classification accuracy

The results of the 6-class classification analysis after removing potentially “bad” trials, as indicated by participants during the verification step of each trial, are reported in Table 6. The reported accuracies represent the grand average balanced accuracies across all participants. Removing trials in which participants responded with either “I did not perform it correctly” or “I performed it correctly but not very well” did not meaningfully improve the average classification accuracies.

**Table 6:** Discarding potentially “bad” data in the 6-class scenarios (%).

**6-Class scenarios with discarding potentially “bad” data**				
**SI type**	**Scenario #1**		**Scenario #2**	
	**All “Did not perform correctly” trials discarded**	**Same number of trials randomly discarded**	**All “Did not perform correctly” and “Performed correctly but not very well” trials discarded**	**Same number of trials randomly discarded**
**SI**_Kin_	60.9 (19.8)	59.7 (20.0)	60.3 (20.1)	59.3 (20.1)
**SI**_noKin_	61.5 (19.3)	60.3 (19.7)	60.8 (19.7)	59.6 (19.9)

#### 3.3.2 Correlation analyses

- Self-evaluation and classification accuracy

No significant correlation was found between the participants' self-evaluation of their task performance and the 6-class classification accuracies (*p* > 0.05).

- KVIQ-10 Scores and classification accuracy

No significant correlation was found between the KVIQ-10 ratings and the 6-class classification accuracies (*p* > 0.05).

- Self-evaluation and KVIQ-10 Scores

No significant correlation was found between the KVIQ-10 ratings and the participants' self-evaluation of their task performance (*p* > 0.05).

## 4 Discussion

### 4.1 Task preferences and difficulty ratings

How practical and user-friendly a BCI is can be significantly impacted by the difficulty of the tasks used for control. Hence, achieving an optimal design is only feasible if we consider not only the accuracy with which the classifier can differentiate the imagery tasks but also how easy it is for the user to perform them. The importance of this is further emphasized when we consider that the target users of active BCI systems are individuals with severe physical disabilities; hence, it is crucial to incorporate tasks that feel intuitive and require minimal training and practice time.

The survey results regarding participants' task preferences suggested that the majority favored the MI of the hands and feet, while fewer participants chose MI of the tongue and the individual SI tasks. However, 13 out of 14 participants did include one of the two SI tasks as one of their top four preferences. The responses to Question 2 further supported the intuitiveness of the SI tasks, as 13 out of 14 participants found the SI tasks preferable to tongue MI. Between the two SI tasks, there was a relatively even split between participants who preferred SI_Kin_ (8) and SI_noKin_ (6).

The difficulty ratings generally supported the survey results. As expected, participants found the REST task to be the easiest to perform among the listed tasks. Following that, the R, L, F, and SI_noKin_ tasks all had similar difficulty ratings (around 2 out of 5), while the T and SI_Kin_ tasks were rated as more challenging (around 2.5 out of 5).

One apparent contradiction in the results is that, despite SI_Kin_ being rated as more difficult on average compared to SI_noKin_, more participants expressed a preference for SI_Kin_.

While it may appear from the task difficulty ratings that participants found the kinesthetic version of SI as challenging as tongue MI, informal feedback from the participants suggested that the perceived difficulty may have been impacted by the effort needed to differentiate the instructions between the two types of SI. Specifically, some participants reported that they rated one or both of the SI tasks as more difficult because they struggled to follow the two different sets of instructions, specifically, focusing on the kinesthetic sensations of movement in one case and avoiding it in the other. This problem would be addressed in a final BCI design by providing only one clear instruction for the SI task.

Although preferences and difficulty ratings are subjective, these results suggest that SI tasks offer an intuitive alternative for users, even if they require slightly greater cognitive effort due to the dual-instruction design used in this study.

### 4.2 EEG classification

#### 4.2.1 2-class scenarios

• **Task vs. REST classification**

For a practical BCI design, any new task introduced to the system should be distinguishable from the rest state (REST). In our study, the mean Task-vs-REST classification accuracies for all tasks exceeded their respective binomial chance thresholds.

Specifically, the R and L tasks exhibited the highest classification accuracy when compared to REST, achieving 85.6% and 87.5% accuracies, respectively. Encouragingly, the accuracy obtained for SI_Kin_ vs. REST (83.1%) was found to be comparable to the accuracies for F vs. REST and T vs. REST (83.6% and 82.7%, respectively). This contradicts the low levels of performance reported in previous studies (Myrden and Chau, 2015; Banville et al., 2017), and indicates that it may be feasible for SI to deliver similar levels of performance in systems with higher numbers of commands. However, the classification accuracy for SI_noKin_ vs. REST was notably lower at 74.1% (Cohen's d = 0.72, *p* = 0.008). This observation suggests that an emphasis on the kinesthetic sensations associated with jaw, tongue, and lip movements during SI generates stronger and/or more distinct patterns of brain activity. Consequently, it may be a more effective version of the task for BCI control.

• **Task vs. Task classification**

To explore the potential use of the SI tasks in binary systems, and before considering their inclusion in multi-class scenarios, we first assessed their differentiability from the other tasks. In this study, the mean Task-vs-Task classification accuracies (including SI_Kin_ vs. SI_noKin_) exceeded their respective binomial chance thresholds.

Based on our results, both SI task variants demonstrated comparable accuracies when classified against the conventional MI tasks. Interestingly, the biggest difference between the two SI tasks was observed when classifying against tongue MI. The accuracy for T vs. SI_noKin_ was 7% higher than for T vs. SI_Kin_ (78.9% compared to 71.8%; Cohen's d = 0.54, *p* = 0.007). This difference can likely be attributed to the fact that for the SI_Kin_ task, participants were specifically asked to focus on the sensations associated with moving the jaws, lips, and tongue. It is likely that similar brain areas are activated in the tongue MI task, leading to reduced task differentiation. As expected given the similarity of the tasks, the classification accuracy for SI_noKin_ vs. SI_Kin_ was the lowest among all 2-class problems, with an average accuracy of 67.6% (though this was still above chance).

The classification accuracies for the remaining task vs. task problems were relatively similar, ranging between 80% and 90%. The highest accuracies were observed for the comparisons of R or L against either T, SI_noKin_, or SI_Kin_, with accuracies ranging between 88.2% and 90.5%. On the other hand, the lowest accuracies were obtained for the comparison of L against R (81.7%) and F against T (82.6%). Furthermore, both SI_Kin_ and SI_noKin_ exhibited slightly higher classification accuracies (2–3%) against F than against T.

These 2-class results reinforce the idea that SI tasks evoke brain activity patterns that are distinct from all conventional MI tasks, with the SI_Kin_ vs. T pairing being the most challenging to separate which is consistent with their shared reliance on articulatory motor representations.

#### 4.2.2 4- and 5-class scenarios

Our results confirm the potential of SI as a viable alternative to traditional MI tasks in multi-class scenarios as well. Specifically, incorporating SI in these scenarios yielded maximum accuracies of 73.7%, and 67.1% for 4-class and 5-class combinations, respectively. In comparison, combinations involving only the conventional tasks for the 4-class and 5-class problems resulted in average accuracies as high as 72.8% and 66.5%, respectively. These findings demonstrate that using SI could allow for the development of 4, and 5-class paradigms that achieve accuracies comparable to the conventional tasks, while potentially offering a more user-friendly approach for some users. A paired *t*-test in the 4-class scenario confirmed that, on average, the accuracies achieved for task combinations including SI were not significantly different from those that did not include SI (diff = 0.9%, *p* = 0.11). Note that such a test was not possible for the 5-class scenario because only one task combination did not include SI.

Our findings from the binary scenarios showed that some pairs of tasks were less distinguishable by the classifier and resulted in lower binary classification accuracies (e.g., T vs. SI_kin_: 71.8%; SI_noKin_ vs. REST: 74.1%) than the other task pairs. Multi-class scenarios appear to follow similar trends, where including both tasks from such pairs in the overall combination resulted in lower performance in comparison to combinations that include only one of the tasks. While the binary classification results were not particularly low for F vs. T (82.6%), including both of these tasks in the multiclass task combinations also seem to produce lower accuracies. *Post hoc* paired t-tests were conducted to compare the average accuracies of combinations that did and did not include one or more of these three task pairs. The results confirmed that accuracies were significantly lower for combinations that included one or more of these task pairs (4-class scenario: Cohen's d = 1.98, *p* = 0.001). In the 5-class scenarios, the average accuracy for task combinations that included the low-accuracy pairs of (SI_Kin_ and T) and (SI _noKin_ and REST) was not significantly different from the average accuracy when those combinations were excluded (p = 0.47). No test was done to determine the effect of including the (F and T) task pair in the 5-class scenario because all task combinations included this pair.

Recognizing a “No-control” or REST state is essential for practical MI-BCI use, as it allows users to pause interaction without continuously performing an imagery task. In our multi-class analyses, all task combinations that included both SI and REST produced mean accuracies that exceeded their binomial chance thresholds. In the 4- and 5-class scenarios, mean accuracies for combinations including SI and REST ranged from 62.0-71.9% and 62.1–66.9%, respectively, indicating that practical multi-class configurations incorporating both SI and REST are feasible.

Overall, the 4- and 5-class results suggest that SI can be incorporated into moderate-sized command sets without degrading system performance. These findings reinforce that SI produces EEG patterns distinct enough from most conventional MI tasks to function as a reliable control option in multi-class MI-BCI settings.

#### 4.2.3 6-class scenarios

The results of the 6-class analysis supports the potential of incorporating a SI task to increase the number of possible commands in MI-BCI. The average accuracies of around 60% are more than three times higher than the theoretical chance level of 16.7% and the statistical significance threshold of 19.8%. While the average accuracies over all participants did not reach the commonly cited threshold of 70% for effective communication (Kübler et al., 2001), they are very close to that level, and it is reasonable to think that acceptable performance may be achievable through further optimization of preprocessing and classification techniques, and with training and practice (with feedback) by the users; indeed, subject training is said to be crucial for acquiring BCI control (Tonin et al., 2022; Kumar et al., 2024). It should also be noted that accuracies exceeding 70% were in fact achieved for 6 out of the 14 participants, with 4 participants reaching accuracies exceeding 80%. These are impressive results for a 6-class problem and indicate the potential for high performance in some individuals, even without training.

Importantly, the accuracy for all participants was significantly higher than the level of statistical significance (for the combination including SI_Kin_: *p* < 0.001, for the combination including SI_noKin_: *p* < 0.001), demonstrating the potential usefulness of both SI tasks. Significant inter-participant variability was observed in the results, which is not unusual for BCI studies. Previous studies suggest that around 15–30% of BCI users cannot generate the EEG patterns required to accurately control a BCI, a phenomenon termed “BCI illiteracy” (Becker et al., 2022; Tibrewal et al., 2022). There are many factors that can contribute to individual variability in EEG classification results, such as differences in cognitive states, as well as anatomical differences in brain structure (Saha and Baumert, 2020). It should be noted that, in this study, participants whose data resulted in lower accuracy levels exhibited low accuracy even for combinations that included only conventional MI tasks, and thus their lower accuracies cannot be attributed to the inclusion of a SI task. Future work should investigate the characteristics of high and low performers to better understand sources of inter-subject variability.

It is also interesting to observe that there was no significant difference between the results obtained from the two SI tasks (Cohen's d = 0.05, *p* = 0.5), indicating that neither task consistently outperformed the other in terms of classification accuracy in the 6-class scenario.

The confusion matrices calculated for the 6-class scenarios provide additional evidence supporting the idea that there are similarities in the brain activity patterns generated by MI of the tongue and the SI tasks, particularly SI_Kin_. The classifier commonly misidentifies the T task as SI_Kin_, or SI_Kin_ as T. Conversely, in the 6-class combination involving SI_noKin_, REST and SI_noKin_ were often confused. Furthermore, consistent with the 2-class results, the F task appears to be more distinct from SI than it is from the T task.

The pattern of confusions observed across the SI and tongue MI tasks is compatible with what is known about the neural substrates underlying these forms of imagery. Studies of imagined or covert speech have highlighted the involvement of classical language areas, including Broca's and Wernicke's regions, as well as premotor, insular, and sensorimotor regions associated with articulatory preparation and motor simulation (Panachakel and Ramakrishnan, 2021; Li et al., 2020). Li et al. further showed that imagined singing can recruit not only articulatory-motor regions but also auditory cortices via an efference-copy-based motor-to-sensory mechanism, paralleling neural processes observed in speech imagery. In contrast, limb MI is known to activate sensorimotor regions in a somatotopic manner. This distinction helps explain why SI is easily differentiated from limb MI tasks but shows some similarity to tongue MI, which also involves articulatory-related representations. This interpretation is consistent with the classification results, where SI consistently achieved accuracies comparable to those of conventional motor imagery tasks across binary and multi-class scenarios. Although we did not directly localize neural sources in this study, this correspondence between expected neural processes and observed confusion patterns supports the view that SI engages a meaningful and reproducible imagery process suitable for MI-BCI integration. We emphasize, however, that these interpretations remain speculative; the present study was not designed to localize neural generators of SI, and our findings speak only to functional separability at the level of classification.

Taken together, the multi-class results illustrate that BCI performance is governed less by the absolute complexity of a task and more by the functional relationships among the imagery processes themselves. Tasks that recruit overlapping neural representations tend to impose performance limits when combined, whereas tasks drawing on more distinct functional systems, such as limb MI and SI, can coexist in larger command sets without substantial loss of accuracy. This suggests that effective multi-class MI-BCI design may benefit from considering the neurophysiological separation between candidate tasks, rather than relying solely on conventions or user intuition. Within this perspective, SI appears to form a clearly distinguishable pattern of activity, supporting its potential value as an additional command option in expanded MI-BCI systems.

Although SI does not correspond to a natural motor action like limb MI, many practical MI-BCI applications require additional self-paced commands such as mode switching, selection, or navigation that do not have intuitive limb-based analogs. SI provides a familiar, easy-to-perform internal action that can serve this role and may be preferable to auxiliary MI tasks (e.g., tongue MI) that are not inherently more “task-relevant.” Unlike reactive BCIs such as SSVEP or P300, SI remains fully voluntary and stimulus-independent, making it suitable for users or contexts where sustained visual attention to external stimuli is not feasible. In this context, SI is best understood as an additional, user-accessible imagery task that can enhance flexibility in MI-BCI design.

### 4.3 Self-evaluation data

The analyses based on participants' subjective ratings - both the KVIQ-10 imagery scores and the trial-level task verification responses - showed no significant correlations with the 6-class classification accuracies. This contrasts with findings such as Vuckovic and Osuagwu (2013), who reported that KVIQ-10 scores predicted BCI performance. In our dataset, neither participants' general imagery ability nor their trial-to-trial self-evaluation of task execution showed evidence of reflecting the quality of the EEG features used for classification. Moreover, because both KVIQ-10 ratings and trial-level verification responses are self-reported, one might expect some internal consistency between them, but no correlation was observed. While these results suggest that subjective self-evaluations did not serve as reliable indicators of imagery quality in this dataset, it is also possible that the absence of significant correlations reflects the limited statistical power of the correlation analysis given the modest sample size and the small number of trials rated as having been performed incorrectly.

There was also no improvement in 6-class accuracy after excluding “bad” trials, but in context this result is understandable. Trials rated as “I did not perform it correctly” were relatively infrequent, suggesting that clear failures to follow instructions were uncommon. Trials rated as “performed correctly but not very well” were more frequent, but likely reflected fluctuations in attention, fatigue, or uncertainty about imagery vividness rather than meaningful changes in the underlying neural patterns. Subjective self-evaluation is coarse and may index perceived difficulty or confidence more than the neural stability of the imagery itself. Participants may simply have limited metacognitive access to the neural quality of their imagined movements or sung sequences. This helps explain why subjective ratings neither correlated with accuracy nor improved classifier performance when used to exclude trials.

Crucially, SI still produced consistently above-chance accuracy in all classification scenarios, indicating that the neural activity associated with SI was sufficiently distinct and reproducible even when participants expressed uncertainty about some individual trials.

### 4.4 Limitations and future work

Although the findings of this study are promising, it was designed as a proof-of-concept to assess the feasibility of SI as a BCI control task, and therefore shares several limitations typical of exploratory BCI research. First, because only healthy participants were included, some unintended muscle activity may have been present and could, in principle, influence classifier performance. However, such residual EMG is unlikely to explain the observed class separability: EMG power is concentrated above the 4–40 Hz bands used in our FBCSP features, and tasks most susceptible to orofacial EMG (SI and tongue MI) did not show systematically higher accuracies relative to either REST or the limb imagery tasks. In addition, the cognitive and neural profiles of individuals with severe motor disabilities may differ significantly from those of healthy participants, which could potentially affect task performance and classifier outcomes when applied to the target population of BCI users. Future studies should include individuals from the target population for validation.

Like many early-stage BCI studies, this investigation included a limited number of participants and was conducted in a controlled lab session with analysis conducted offline. While this allows for consistency and control, it does not fully reflect real-world conditions, where external factors and environmental variability can affect both user performance and the practicality of BCI systems. As such, further research involving larger, more diverse samples and conducted in online, ecologically valid settings is necessary to evaluate the generalizability and real-world applicability of the findings.

Furthermore, due to the offline and exploratory nature of the study, participants completed a single experimental session and received no training or feedback regarding their performance of the tasks. Future studies should involve investigating the performance of individuals while undergoing training in the use of the BCI over multiple sessions, which would provide valuable insights regarding the true potential of SI as a control task, and how it compares to conventional MI tasks in practical situations over time.

In order to gain a clearer understanding and assess the two types of SI (SI_Kin_ and SI_noKin_), it would be beneficial to recruit two separate groups of participants. Each group would solely engage in one type of SI, thus minimizing the potential for the “crosstalk” between the two SI tasks and reducing the challenge for the participants of properly executing the two sets of instructions.

Future work should explore how different preprocessing pipelines, including more advanced artifact-removal methods, affect SI-based MI-BCI performance and task separability.

## 5 Conclusion

Our investigations indicate that singing imagery could provide a robust and potentially more intuitive alternative for conventional motor tasks in designing active BCIs. Moreover, the results suggest that it may be feasible to use singing imagery for increasing the number of distinguishable commands to six and achieve average accuracies around 60%. An increase in the number of commands would lead to an enhancement in the practicality and functionality of conventional active BCI systems. Future investigations on potential usability of singing imagery in active BCI would benefit from exploring this task in online and potential real-world scenarios, and with individuals from the target population of BCI users.

## Data Availability

The datasets presented in this article are not readily available because participants did not consent to having their data shared beyond the research team. Requests to access the datasets should be directed to sd.power@mun.ca.
